# New Treatment Horizons in Uveal and Cutaneous Melanoma

**DOI:** 10.3390/life13081666

**Published:** 2023-07-31

**Authors:** Daciana Elena Brănişteanu, Elena Porumb-Andrese, Vlad Porumb, Alexandra Stărică, Andreea Dana Moraru, Alin Codruț Nicolescu, Mihail Zemba, Cătălina Ioana Brănişteanu, George Brănişteanu, Daniel Constantin Brănişteanu

**Affiliations:** 1 Department of Medical Specialties (III)-Dermatology, Faculty of Medicine, “Grigore T. Popa” University of Medicine and Pharmacy, 700115 Iasi, Romania; daciana.branisteanu@umfiasi.ro; 2 Railway Clinical Hospital, 700506 Iasi, Romania; dbranisteanu@yahoo.com; 3 Department of Surgery, Faculty of Medicine, “Grigore T. Popa” University of Medicine and Pharmacy, 700115 Iasi, Romania; vlad.porumb@umfiasi.ro; 4 Military Emergency Clinical Hospital “Dr. Iacob Czihac”, 700506 Iasi, Romania; 5 Department of Ophthalmology, Faculty of Medicine, “Grigore T. Popa” University of Medicine and Pharmacy, 700115 Iasi, Romania; andreea_moraru10@yahoo.com; 6 “Agrippa Ionescu” Emergency Clinical Hospital, 011773 Bucharest, Romania; nicolescualin66@yahoo.com; 7 Department of Ophthalmology, “Carol Davila” University of Medicine and Pharmacy, 020021 Bucharest, Romania; mhlzmb@yahoo.com; 8 “Grigore T. Popa” University of Medicine and Pharmacy, 700115 Iasi, Romania; branisteanucatalina@yahoo.com (C.I.B.); office_george@yahoo.com (G.B.)

**Keywords:** uveal melanoma, metastatic melanoma, cutaneous melanoma, genetic mutations, chromosome aberrations

## Abstract

Melanoma is a complex and heterogeneous malignant tumor with distinct genetic characteristics and therapeutic challenges in both cutaneous melanoma (CM) and uveal melanoma (UM). This review explores the underlying molecular features and genetic alterations in these melanoma subtypes, highlighting the importance of employing specific model systems tailored to their unique profiles for the development of targeted therapies. Over the past decade, significant progress has been made in unraveling the molecular and genetic characteristics of CM and UM, leading to notable advancements in treatment options. Genetic mutations in the mitogen-activated protein kinase (*MAPK*) pathway drive CM, while UM is characterized by mutations in genes like *GNAQ*, *GNA11*, *BAP1*, *EIF1AX*, and *SF3B1*. Chromosomal aberrations, including monosomy 3 in UM and monosomy 10 in CM, play significant roles in tumorigenesis. Immune cell infiltration differs between CM and UM, impacting prognosis. Therapeutic advancements targeting these genetic alterations, including oncolytic viruses and immunotherapies, have shown promise in preclinical and clinical studies. Oncolytic viruses selectively infect malignant cells, inducing oncolysis and activating antitumor immune responses. Talimogene laherparepvec (T-VEC) is an FDA-approved oncolytic virus for CM treatment, and other oncolytic viruses, such as coxsackieviruses and HF-10, are being investigated. Furthermore, combining oncolytic viruses with immunotherapies, such as CAR-T cell therapy, holds great potential. Understanding the intrinsic molecular features of melanoma and their role in shaping novel therapeutic approaches provides insights into targeted interventions and paves the way for more effective treatments for CM and UM.

## 1. Introduction

Many hypotheses deliberate why cells with similar embryonic origins and biological functions (i.e., melanin production) undergo diverse tumor transformation pathways. Significant knowledge concerning the features of cutaneous and uveal melanoma has been acquired in the last decade, including molecular and genetic characteristics, primary tumor treatment, and metastatic disease approach.

Genetic alterations play a crucial role in the development and progression of uveal and cutaneous melanoma and these features have led to significant advancements in treatment options for these aggressive forms of cancer. Thus, more preclinical studies and testing of the active substances of newly emerging therapies should utilize specific model systems for uveal and cutaneous melanoma due to their unique genetic characteristics [1].

This review delineates the intrinsic molecular characteristics of melanoma and demonstrates how these features provide a basis for new therapies such as viral oncolysis and immunotherapies, as standalone treatments or in conjunction with each other.

## 2. Genetic Profiles

CM and UM are genetically distinct tumors. Defects in proteins involved in the mitogen-activated protein kinase (*MAPK*) pathway are found in most CM cases. This is a crucial intracellular signaling system that plays a role in cell growth, division, and survival [2]. Although multiple mechanisms can activate the *MAPK* pathway ontogenically, the most prevalent is a constitutively activated mutant *BRAF* kinase. *BRAF* kinase mutations are encountered in 40–60% of the cases of CM. *BRAF*-mutated melanoma has different clinical characteristics and is associated with a more aggressive bioactivity than *BRAF* wild-type (WT) melanoma. *BRAF*-mutated melanoma may be connected to a significantly shorter life expectancy and poor prognostic indicators; however, this is currently under investigation [3,4,5]. Mutated *NRAS* is the second most frequent *MAPK* pathway aberration in CM, occurring in 15–30% of patients [6,7]. CM with mutations in the stem cell factor receptor tyrosine kinase gene (*KIT*) is an uncommon subtype that occurs in around 20% of mucosal, acral, and chronically sun-damaged areas [8]. The findings that a mutation in *BRAF* kinase causes many of the CM has led to the development of the selective inhibitors of the *BRAF* V600-mutated kinase (vemurafenib, dabrafenib, and encorafenib) as well as inhibitors of the downstream *MEK* kinase (trametinib, cobimetinib, and binimetinib). Although individuals with the *BRAF* V600E or V600K mutation have a high response rate to *BRAF* treatment, most patients will develop acquired resistance. When compared to *BRAF* monotherapy, a combination of *BRAF* and MEK inhibitors seems to be more effective in preventing acquired resistance [9].

*GNA11*, *GNAQ*, *BAP1*, *EIF1AX*, and *SF3B1* are typically the affected genes in UM [2]. *BAP1* is a tumor-suppressor gene on chromosome 3, which is mutated in 47% of those with UM. Inactivating mutations in the isolated viable *BAP1* gene cause [10] UM in BAP1 germ-line mutants, which is like the common loss of chromosome 3 seen in high-risk sporadic disorders [11]. Patients with UM have an 11% higher risk of secondary malignancies, including cutaneous melanoma, compared to the general population, which can be related to germline *BAP1* mutations [12]. *GNA11* or *GNAQ* mutations occur in more than 90% of UM and these mutations stimulate signaling between G-protein-coupled receptors and downstream effectors, as well as upregulate *MAPK* pathway signaling [13]. In most uveal melanomas, these alterations are strictly exclusive and are recognized as an early event in the pathogenesis of UM. *GNAQ* and *GNA11* mutations were not correlated with a worse prognosis or the development of metastatic tumors [14,15]. Primary UM has been separated into four clinically meaningful molecular categories, each with a distinct metastatic rate and prognosis [13]. Tumors belonging to classes 1A and 1B have distinct melanocyte phenotypes, implying mutations in either *EIF1AX* or *SF3B1*. with an alteration of chromosome 3, Class 1A presumes a lower metastatic rate than 1B. Class 2 UM is distinguished by chromosome 3 monosomy, *BAP1* expression abnormalities, and ubiquitous DNA methylation, and is associated with a high metastatic risk. Based on chromosome 8q copy number mutations, RNA expression patterns, and cellular pathway activity profiles, classes 2A and 2B were defined [16]. When compared to Class 2A, Class 2B has an increased metastatic rate [17]. Since most UM are generated by mutations in *GNAQ* or *GNA11*, studies have been directed into treatments that target the downstream effectors of these pathways such as *MEK*, *Akt*, and protein kinase C (*PKC*). Unfortunately, the findings were unsatisfactory, with response rates averaging less than 10% [18]. Epigenetic dysregulation could be a successful innovative target in UM. Somatic mutations throughout the tumor suppressor gene *BAP1* were associated with metastatic behavior, as previously disclosed. The deletion of *BAP1* gene appears to make UM cell lines more susceptible to histone deacetylase (HDAC) inhibitor therapy. Both in vivo and in vitro, HDAC causes a G1 cell cycle arrest with an enhanced cyclin D1, a decreased cell proliferation, growth inhibition, and apoptosis activation in UM [19,20].

Given the fact that the relationship between histone acetylation and deacetylation is altered in various types of cancers, therapy with HDAC inhibitors may be effective for both UM and CM. This balance determines the extent of histone acetylation and thus represents a crucial factor in gene expression regulation [21]. While acetylation by histone acetyltransferases (HAT) is correlated to gene transcription, histone deacetylation by HDAC is involved in gene suppression. HDAC inhibition was found to suppress cell growth and division [22].

## 3. Chromosomal Aberrations

The deletion of one of the two copies of chromosome 3 (monosomy 3) is the most common chromosomal abnormality encountered in UM. Monosomy 3 is found in about 50% of all cases [23] and appears to be a chromosomal aberration unique to UM, as it is rarely seen in CM or other forms of cancer [24]. Several studies have demonstrated a significant relationship between monosomy 3 and the onset of metastatic disease [25]. Monosomy 3 is also associated with epithelioid cytology, closed vascular patterns, a large tumor diameter, and ciliary body involvement in clinical and histopathological examinations [26]. In addition, monosomy 3 is considered to appear early in the tumorigenesis process because it frequently occurs in conjunction with all other known chromosomal alterations [27].

A gain of 8q (+8q) is identified in around 40% of UM cases and is a major independent prognostic marker for poor survival [25]. It is usually observed in conjunction with monosomy 3, either as +8q or as isodisomy 8q, and this combination has been strongly correlated with metastatic tumors [25,28]. Chromosome 3 and 8 aberrations are more common in ciliary body UM, whereas alterations on the long arm of chromosome 8 are more common in choroid UM [25,26,27]. While chromosome 8q aberrations were shown to be associated with a large tumor diameter in one study [29], a univariate analysis revealed no significant relationship between the gain of 8q and the metastatic phenotype. A gain of 8q is also frequently detected in various copy numbers in UM, suggesting that it is a late event following the initiation of monosomy 3 [30].

Although chromosome 8q gain is encountered in 25% of CM, the concomitant occurrence of monosomy 3 and 8q gain, as observed in UM, is uncommon in CM [31,32].

The loss of 1p36 in combination with monosomy 3 has been proven to be a relevant prognostic factor: these aberrations occurring together have shown a stronger correlation with poor survival than monosomy 3 or the loss of 1p36 alone (although—1p36 alone is not of prognostic value) [33]. The frequent deleted sites on chromosome 1 were identified to range between 1p34.3 and 36.2 [34].

Chromosome 6 changes occur in both UM and CM, but they have a lower prognostic value as compared to monosomy 3 or a gain of 8q in UM [24,25]. Among all these alterations, the gain of DNA material on the short arm of chromosome 6 (+6p) has been identified in 25–29% of UM and is associated with spindle cell morphology and a lower probability of evolution to metastatic disease [24,27,35]. The concomitant occurrence of +6p and -3 is an exceptional event. The deletion of DNA material on the long arm of chromosome 6 (-6q), occurring in 25–38% of UM, might be another late event in the carcinogenesis process and is associated with a worse prognosis [24,26,35,36]. Other chromosome abnormalities, such as loss of 9p, loss of chromosome 10, loss of 11q23–q25, and gain of chromosomes 7 and 10, have been rarely described [25,26,36], but their involvement in the tumorigenesis process and metastatic disease development in UM has yet to be determined.

In comparison to UM, CM exhibits a more complex karyotype. Monosomy 10 is the most common chromosomal abnormality in CM individuals. Monosomy 10 is identified in 60% of CM cases, which appears to be considerably more prevalent as compared to UM, where monosomy 10 is documented in only 27% of the cases. Other chromosomal abnormalities associated with CM include the loss of 1p, 4, 5, 6q, 9p, 11q, 12q, 14, 15, 16, 17p, 21, and chromosome 22 and a gain of DNA material on chromosomes 1q, 7, 18, and 20 [24].

Rearrangements of the distal region of chromosome 1’s short arm, resulting in a loss or gain of 1p, have been documented in 28% and 33% of CM. Because they include the *NRAS* and *AKT3* genes, some areas of chromosome 1 are of relevance. *NRAS* is found on chromosome 1p13 and has been demonstrated to be triggered by a mutation in 15–25% of CM [37]. *NRAS* is thought to be involved as well in the *MAP*-kinase pathway. The *MAP*-kinase pathway is initiated by the activation of the *NRAS* gene, ultimately leading to cellular proliferation. *NRAS* is binding and activates the lipid kinase phosphoinositide-3 kinase (*PI3K*), which promotes the *AKT* pathway activation and prevents apoptosis [38]. In 40–67% of CM, a direct activating mutation of the *AKT3* gene on 1q44 is identified [39]. *PTEN* loss can lead to the selective activation of *AKT* in CM [40], while the overexpression of AKT3 makes cells less sensitive to apoptotic stimuli. *NRAS* mutations are quite uncommon in UM, according to many studies [41,42].

In total, 66% of CM displayed chromosome 6 abnormalities, with +6p occurring in 24% and -6q observed in 42% [24]. The 6q10–q27 region presents, by far, the most rearrangements because of the deletion, translocation, or development of an isochromosome of its short arm. The segment on the short arm of chromosome 6 that is often altered spans 6p21 to 6p25 and is characterized by DNA material gain. Both +6p and +6q are prevalent in UM, as previously stated. However, the prognostic value of these abnormalities is less significant than in CM [24,25].

The gain of DNA material on both arms of chromosome 7 is reported in 36% of CM. Somatic mutations in the 7q34 region, where the *BRAF* gene is found, are the most frequently reported. Activating mutations in *BRAF* are detected in up to 60–70% of CM [43]. The *BRAF* gene encodes a kinase that is essential in the *MAP*-kinase pathway and is thought to be involved in the constitutive activation of the pathway and cell proliferation when mutated. More than 90% of all *BRAF* mutations appear to be caused by a single alteration (p.V600E) [44]. The identical mutation is also identified in 80% of benign naevi, suggesting that it might be a precondition to melanoma genesis [45] and implicated in later phases of tumor growth [45]. *BRAF* mutations are missing in UM pathology [41,46]. However, a small study has identified *BRAF* mutations in 48% of iris melanoma [46]. Chromosome 9 abnormalities exhibiting deletions of the short arm, -9p10–24 (37% of CM), or the long arm, +9q22–34 (15% of CM) have been reported. *CDKN2A*, which is located on 9p21, is one of the well-studied genes in CM. The two encoding tumor suppressor genes p16 and p14 are inhibited by inactivating mutations or deletions. Both genes were previously associated with significant CM susceptibility and were found in 30–80% of familial CM cases [47]. On the contrary, these mutations are infrequently identified in sporadic CM or UM [48].

## 4. Immune System Implications

Various malignancy types feature different distributions of immune cells. Over the last few decades, the adaptive immune response’s function in tumor growth has garnered considerable attention in CM. The presence of CD3+CD8+ lymphocytes in both the tumor and the stroma, specifically activated (HLA-DR expressing) CD8+ T cells, was associated with disease-specific survival in primary CM [49]. Moreover, the role of macrophages has also been evaluated. The macrophages that support an appropriate antitumor response (M1) and the macrophages that stimulate tumor development (M2) are two primary subtypes of macrophages. The M1-recruited macrophages transition to the M2 phenotype early in the development of CM, increasing tumor proliferation and metastasis. In contrast to CM, a significant degree of immune cell infiltration of UM, such as lymphocytes and macrophages, is correlated with a poor prognosis [50]. Increased lymphocytes in tumor cells may be causing an increase in inflammatory mediators’ synthesis, resulting in a tumor-promoting inflammatory environment. Phagocytic activity, tissue remodeling, tumor development, and angiogenesis are promoted by M2 phenotype macrophages [51].

UM does not display any ultraviolet mutation signature [52].

## 5. Diagnostic

The clinical assessment of suspicious lesions is a crucial component of CM and UM diagnoses. Dermatologists use clinical evaluation to diagnose CM and reserve excisional biopsy for tumors with uncertain etiology. The iris melanoma can be detected earlier, at a routine slit-lamp evaluation, or noticed by the patient themselves due to the iris color heterogeneity. On the contrary, both choroidal and ciliary body melanomas are diagnosed much later at a funduscopic examination with dilated pupils completed by an ultrasound evaluation as they can be asymptomatic for a long period. Tumor growth can cause in such cases heterogenous manifestations, ranging from mild visual field abnormalities to central visual loss, due to accompanying retinal detachment or massive extraocular tumor extension [53].

Early signs of melanoma presence and growth are easily omitted by the patient. Nevertheless, the tumor thickness and diameter at the time of diagnosis are considered to have a major impact on the overall survival rate in both tumors. As a result, practitioners must remain focused on the early diagnoses of CM and UM. Early detection resulted in an average CM thickness decrease of 0.76 mm at the time of diagnosis, associated with a 90% overall 10-year survival rate [54]. UM with a diameter of less than 4 mm have an 84% 5-year survival rate. Medium-sized UM (4–8 mm in diameter) has a 5-year survival rate of 68%, while large UM (≥8 mm in diameter) has a 5-year survival rate of 47% [55]. Patients with metastatic disease from both CM and UM have a poor life expectancy of 2–7 months [56,57].

## 6. Primary Tumor Treatment

CM and UM require very different therapeutic approaches [58]. An excisional biopsy of the main tumor is recommended as the primary treatment for CM. Wide local excision is performed, with the requirement of different negative margins depending on the tumor thickness [59]. Additionally, the use of Mohs micrographic surgery (MMS), a specialized surgical excision technique which promotes tissue sparing and provides optimal margin control through a complete evaluation of both the peripheral and deep margin for the treatment of CM, has been gaining the same widespread attention as standard wide local excision. Even though there is growing evidence demonstrating similar or improved cure rates when compared to standard excisional biopsy, MMS is not recommended for the routine surgical management of CM [60,61].

Regarding the radical surgical treatment of the UM enucleation of the eye, which was once considered the gold standard in the treatment of intraocular tumors, is still recommended in cases of large UM, tumors that are unresponsive or recurrent to conservative treatments, and total visual loss due to severe complications [62,63].

Over time, the treatment of primary UM has constantly improved and different radiation techniques such as brachytherapy, proton beam radiotherapy, and stereotactic radiation therapy using CyberKnife, Gamma Knife, or a linear accelerator have successfully replaced enucleation in selected cases [51,63,64]. Currently, conservative treatment using a radioactive plaque temporarily sutured on the sclera adjacent to the tumor is one of the most effective and preferred methods for controlling medium-sized UM [65]. Also, eyes with extensive orbital tumor development are now treated more conservatively, avoiding orbital exenteration with a combination of the surgical enucleation of the eye and local radiation therapy [66].

Other conservative treatment methods, less widespread and with more limited indications, include watchful waiting in the case of inactive small uveal lesions, direct laser photocoagulation—which has been abandoned in many centers due to the modest tumor control and the increased rate of recurrence—transpupillary thermotherapy for the treatment of small UM, and photodynamic therapy, which has been approved by the FDA for the selective treatment of choroidal neovascularization secondary to various conditions [67].

The recent past has witnessed unprecedented clinical progress in the treatment of advanced CM with multiple therapeutic options and a longer, more durable survival in this type of cancer [68]. Adjuvant and neoadjuvant therapy have evolved to benefit individuals who are most at risk of disease recurrence following surgical excision. In both the adjuvant and neoadjuvant settings, as CM has historically been considered radioresistant, early antitumor chemotherapeutic and biochemical agents are giving way to novel immune therapies, mutation-specific targeted therapies, and oncolytic vaccinations, which are revolutionizing the treatment of CM. The combination of these systemic medications with surgical treatment has been found to improve overall survival. In the adjuvant treatment of advanced melanoma, targeted treatments are considered first-line therapy. These monoclonal antibodies specifically target mutant proteins in the *MAPK* pathway induced by common *BRAF* and *MEK* gene mutations that result in uncontrolled cell proliferation [69].

Other noteworthy therapies include immunotherapeutic drugs used to treat CM, which target checkpoint molecules that are bypassed by the common mutations detected in melanoma cells. Cytotoxic T-lymphocyte antigen 4 (CTLA4) inhibitors (ipilimumab), programmed cell death-1 (PD-1) pathway inhibitors (pembrolizumab, nivolumab), and high-dose interferon-alpha-2b (IFN-alpha-2b; HDI) are several of the most promising types [70,71,72].

Advances in immune checkpoint therapy have led to an improvement in overall survival for patients with advanced melanoma, and combining immunotherapeutic agents with different mechanisms of action may also enhance efficacy [73].

Oncolytic virotherapy (OV) is a new approach in cancer therapy that uses native or modified viruses that selectively infect malignant cells. There are several mechanisms described: virus-mediated lysis, viruses that promote immunogenic cell death, inflammatory response, and localized cytokine production or the disruption of tumor vessels, potentially facilitating immune cell migration into the tumor microenvironment (TME) (for example, HSV-1). Talimogene laherparepvec (T-VEC) is the first OV approved for melanoma treatment, a first-class recombinant type 1HSV [74].

Other oncolytic viruses such as coxsackieviruses have also been shown to have promising potential as effective oncolytic therapies for melanoma, as well as reovirus, rigvir, poxvirus, or adenovirus [75,76].

Also, recent studies have shown the utility of recombining OV and CAR-T (chimeric antigen receptor) cell therapy in mice. The data highlight that the stimulation of this combination could provide synergistic interactions in mouse melanoma tumor model [77].

HF10 is a non-neuroinvasive HSV-1 virus with naturally occurring deletions and insertions that decrease the potential in non-tumor cells while allowing for active proliferation in tumor cells. A phase I clinical trial in patients with refractory superficial cancers and melanoma was conducted at the University of Pittsburgh in the United States. This trial evaluated the tolerability and efficacy of HF10 therapy in 26 patients, including HSV seropositive and seronegative patients, with refractory superficial cancers and melanoma. The reduction in tumor size in some patients ranged from 30 to 61%. Interestingly, one patient showed a pathological complete response after 4 months of treatment [78].

A new approach is to enhance the efficiency of OVs by genetically encoding one or more tumor-associated antigens and neoantigens (TAAs) into the OV genome. Another strategy to enhance OV is the coating of OVs with specifically designed tumor epitope peptides. For example, the intratumoral administration of adenoviruses coated with modified tumor epitope peptides (PeptiCRAd) induces systemic anticancer immunity in mouse and humanized mouse cancer models of melanoma [79,80].

OVs possess the capability of selectively inducing oncolysis, as well as drawing in immune system cells, activating them, and consequently instigating both innate and adaptive antitumor responses, with minimal systemic effects [81]. The viruses can generate “danger signals” that create a less immune-tolerant tumor microenvironment, act as carriers for the expression of inflammatory and immunomodulatory cytokines, and present antigens associated with the tumors. The effectiveness of treatments utilizing OVs was first demonstrated through the application of a genetically engineered herpes simplex virus known as talimogene laherparepvec (T-VEC), in the management of CM [82].

The herpes simplex virus presents an appealing prospect for an OV in melanoma due to its large genome containing several non-essential genes that can be eliminated to reduce pathogenicity, thereby enabling the insertion of genes of interest [83].

Talimogene laherparepvec (T-VEC) is the first and still stands as the sole oncolytic virus authorized by the FDA for the management of CM, including in stages III and IV melanoma [84].

Positive outcomes were observed in phases I, II, and III clinical trials involving the administration of T-VEC for the management of melanoma [82]. A randomized clinical trial represented the first instance of demonstrating the advantageous therapeutic potential of OV for individuals with advanced or unresectable CM [83].

While its use as a neoadjuvant therapy is emerging, further data are necessary to establish its efficacy. Oncolytic viruses are expected to have long-term application in CM treatment, with T-VEC specifically predicted to maintain its function in managing patients with easily reachable cutaneous lesions, both for local containment and to induce a synergistically antitumor immune response, as part of combination therapies.

Currently, several new viral vectors, such as coxsackieviruses, HF-10, adenovirus, reovirus, echovirus, and Newcastle disease virus, are being actively developed and investigated for their effectiveness in targeting CM, with varying degrees of success [84].

In an in vitro investigation involving HF10, it was discovered that an infection with this virus and a strain of HSV-1 that has undergone spontaneous mutation, resulting in the deletion of certain viral genes, had significant cytolytic effects on murine and human melanoma tumor cells. In that same study, HF10 was administered intratumorally to immunocompetent mice with advanced melanomas. The findings showed a decrease in tumor growth in both injected and non-injected tumors, indicating direct oncolysis as well as the induction of a systemic antitumor immune response [85,86].

In preclinical investigations, coxsackievirus A21 displayed oncolytic activity in melanoma cells while maintaining low viral pathogenicity and tolerability [82]. The clinical assessment of CVA21, a commercial variant of coxsackievirus A21, was carried out in phases I and II on patients with advanced and unresectable melanoma who were treated with an intratumoral administration of the virus for 15 weeks. The outcomes demonstrated that the treatment was generally well-tolerated, with low-grade reactions, and resulted in complete therapeutic responses and an acceptable safety profile [87].

In a phase II trial, the oncolytic properties of a derived OV reovirus were examined in patients with metastatic melanoma who received the therapy through intravenous injections. All the patients tolerated the injections well, and in a few cases, viral replication was noticeable when analyzing post-treatment biopsy samples. However, the research did not obtain objectively identified responses or achieve its primary efficacy objective. Despite this, the trial data support using reovirus in combination with other therapies for treating malignant melanoma [88].

Currently, several oncolytic viruses are being tested as well for the treatment of uveal melanoma. The oncolytic potential of the present ECHO-7 virus strain has been observed in several malignancies, including melanoma cell lines [89]. Therefore, the experiments were extended to examine the effect on the cell viability of cytolytic ECHO-7 virus strain on UM cell lines, and out of the seven cell lines tested, only 3 UM cell lines were able to be successfully propagated in the laboratory [90].

It is also noteworthy that phase I clinical trials have evaluated the use of oncolytic adenovirus ICOVIR-5 for the treatment of cutaneous and uveal melanoma. Although the virus was able to reach the tumor, a single injection did not result in any observed effects on tumor regression. This suggests that a systematic administration of the virus over a longer duration should be investigated [91,92].

**Figure 1:**
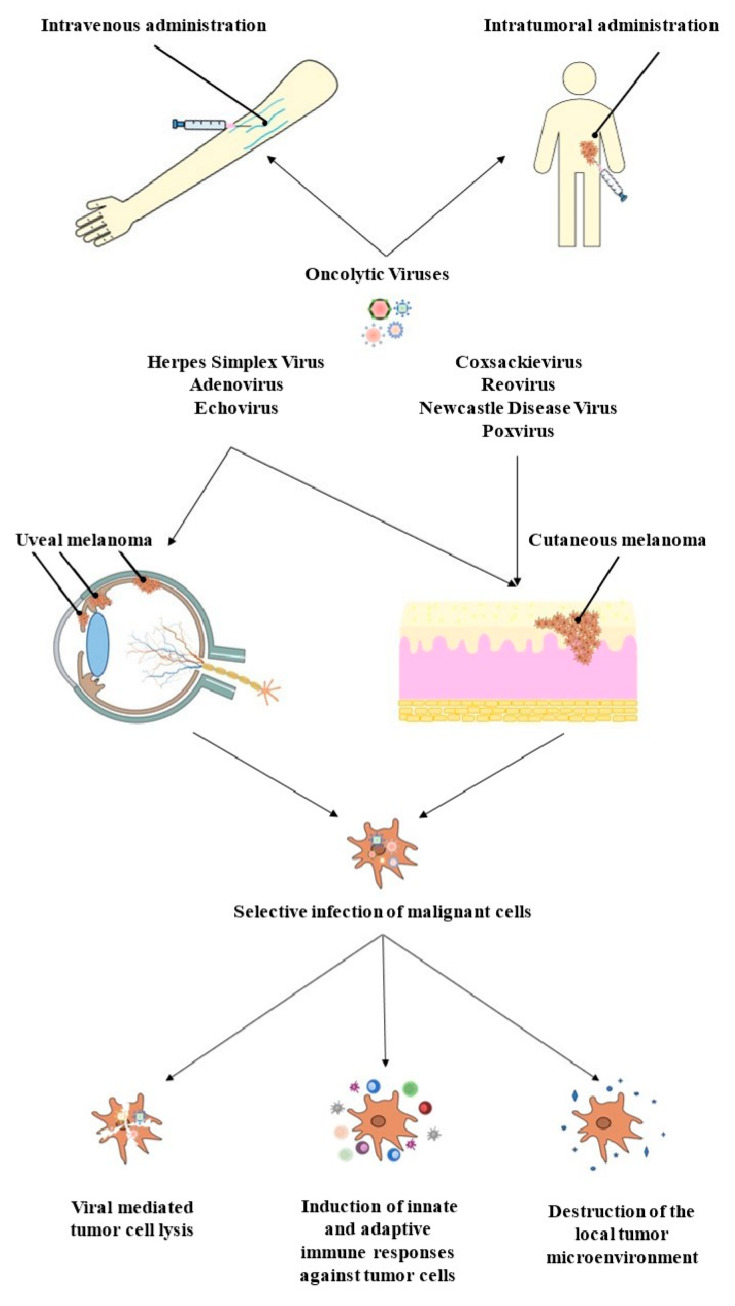
Newcastle disease virus, poxvirus, reovirus, and coxsackie virus are OVs which have demonstrated oncolytic properties against cutaneous melanoma. Herpes virus, adenovirus or echovirus-based OVs, have been shown to possess an anti-tumor effect on both uveal and cutaneous melanoma cells. OVs can be administered intravenously or through a direct intratumoral injection. OVs can selectively target cancerous cells and tissues and induce tumor cell lysis, activate innate and adaptive immune system responses, and damage the tumor microenvironment.

Taking into consideration the success that HSV already has as a treatment for CM, the oncolytic potential has also been tested in 3D uveal melanoma cell spheroids. According to the results, the HSV-1 virus exhibits oncolytic potential in certain melanoma cell lines, but it also stimulates the growth of other melanoma cells (Figure 1).

## 7. Metastatic Disease

Both tumors display a strong tendency to metastasize, although the mode of spreading is different [93]. The development of metastatic disease is a significant predictor of the clinical course and survival in both CM and UM [2].

CM tends to spread mainly via the lymphatic system; however, hematogenous dissemination has been reported as well. Although all organs can be implicated, the skin (13–38%), the lungs (18–36%), distant lymph nodes (5–34%), distant subcutaneous tissues (32%), the brain (2–20%), and the bones (4–17%) are the most prevalent sites for distant CM metastases. Approximately 14–20% of people will develop liver metastases [94].

The uveal tract is devoid of any lymphatics, so ocular melanoma spreads mainly via the bloodstream (hematogenous). The liver is the most predominant metastatic region (80–90% of cases), followed by the lung (24%) and the bones (16%) [95]. Regardless of enucleation or eye-sparing radiation therapies, 45% of UM patients die from metastatic disease [87]. This has led to hypotheses about the presence of micrometastasis in the early stages of the disease, misdiagnosed for years before becoming clinically detectable macrometastasis [96]. The duration of the state of dormancy, as well as the triggers for metastatic development, are still unknown. Tumors as small as 1.0 mm do metastasize, highlighting the necessity for highly specific and sensitive prognostic markers to determine which patients are at risk of developing metastasis. Early metastatic disease and shorter survival are mainly associated with the age of over 60 years, a maximum basal tumor diameter over 18 mm, an epithelioid tumor cell type, and closed vascular patterns [97,98].

Regarding CM, an increasing Breslow tumor thicknesses, invasion level, an older age, male gender, head/neck or trunk primary tumor anatomic site, the number of metastatic lymph nodes, and ulceration on histopathological examination are independent significant prognostic factors of early metastasis. The TNM-staging system summarizes these aspects into a single staging system. This system is based on the tumor stage at the time of diagnosis, which has been proven to be the most significant prognostic factor in CM and is now commonly utilized for prognosis and clinical decisions. Integrin expression levels of circulating plasma exosomes isolated from CM and UM, for instance, are a prognostic factor for determining future metastatic sites. Furthermore, chemokine receptors are expressed in a wide range of malignancies, and their ligands are present in the organs with the highest frequency of metastasis. Chemokine receptors may have an impact on overall survival in patients and could be used as a therapy target [99].

Overexpression of *c-Met*, a hepatocyte growth factor (HGF) receptor, is linked to tumor growth and metastasis in CM. In vitro, suppressing HGF-induced *c-Met* proliferation inhibited the migration and invasion of melanoma cell lines [100]. In UM, *c-Met* accelerates tumor growth and facilitates tumor invasion. The presence of *c-Met* in primary UM increases the possibility of hepatic metastasis development. Cabozantinib is a tyrosine kinase inhibitor that targets the receptors for *MET*, *AXL*, and *VEGF* (vascular endothelial growth factor). It inhibits HGF-induced migration and invasion in CM cells, and it has been demonstrated to inhibit liver metastasis development in a UM xenograft model [101,102,103].

Insulin-like growth factor-1 (IGF-1) is a polypeptide hormone that stimulates tissue growth and has been associated with the development of a variety of tumors, including CM. The blood IGF-1 level served as a prospective prognostic biomarker for metastatic tumor development in both CM and UM. Remarkably, whereas metastatic UM patients reported lower IGF-1 serum concentrations than healthy controls, metastatic CM patients exhibited greater IGF-1 serum levels [104,105]. In UM, an increased insulin-like growth factor 1 receptor (IGF-1R) expression was detected in liver metastasis and was correlated with mortality in the context of metastatic disease. In both CM and UM, the IGF/IGF-1R axis has been a target for novel therapy combinations. Considering that IGF plays a role in both primary and acquired treatment resistance, IGF-targeting medications have been administered in combination with other therapeutic approaches in CM [106,107].

In various forms of cancer, hypoxia-inducible factor (HIF) plays a significant role in carcinogenesis and metastatic dissemination. It plays a crucial function in the development of skin melanoma from melanocytes. HIF activity is upregulated in melanoma even at normal oxygen levels, boosting tumor cell infiltration into surrounding tissues and preserving adequate blood supply. *FBXO22* has recently been suggested as a potential new therapy approach for CM since it is thought to modulate HIF expression. In UM, it was reported that a relative hypoxic activity identified the subtypes, regardless of chromosome 3 status [49,108].

There exists a demand for further treatment options for metastatic melanoma beyond FDA-approved anti-PD1 antibodies, which are only sanctioned for adjuvant therapy in stage III or resected stage IV melanoma [90].

The evaluation of the immune system’s role in tumorigenesis in the latest research resulted in the TIL (tumor-infiltrating lymphocyte) clinical success of adoptive cell therapy (ACT) in the treatment of CM. Due to its revolutionary status, TIL treatment has been applied to various malignancies, including UM, although its efficacy has been documented mainly in metastatic cutaneous melanoma [109,110,111,112].

Treatment with chimeric antigen receptor (CAR) T cells is another cell-based therapy that might be attractive. Both the United States and the European Union have approved two CAR-T cell models that target CD19 in hematological malignancies. c-Met is a target antigen in one of the pilot trials actively recruiting melanoma patients now. Considering that c-Met is associated with both CM and UM, this could be a promising therapeutic approach for both types of melanomas [2].

The FDA and EMA have recently approved tebentafusp for the treatment of patients with metastatic UM and HLA-A*02:01 positivity, which is encountered in approximately 45% of the Caucasian population. The bispecific fusion protein targets gp100 through a T cell receptor (TCR) binding domain and a CD3 T-cell engaging domain. In pivotal clinical studies, the drug demonstrated a 1-year overall survival in 73% of treated patients versus 59% in the control group and a progression-free survival of 31% versus 19% in the control group at 6 months [113,114].

## 8. Future Directions

CM presents significant challenges in its management, as traditional chemotherapy exhibits a limited response rate. Immunotherapies have shown promise, but there is a pressing need for more effective treatments. Molecularly targeted therapies, including FDA-approved tyrosine kinase inhibitors (TKIs), have demonstrated initial efficacy [115]. However, prolonged use often leads to acquired resistance through genetic mutations and alternative mechanisms in melanoma tumors, undermining their long-term effectiveness. Efforts to enhance treatment options focus on understanding drug resistance mechanisms, particularly “phenotype switching”, and investigating novel combination therapies that target both fast- and slow-proliferating cells [116]. Overcoming drug resistance is crucial to improve outcomes and prolong survival for melanoma patients, urging the continued exploration of innovative approaches and new therapeutic targets (Table 1) [117].

Among these promising biomarkers, nicotinamide N-methyltransferase (*NNMT*) has emerged as a key player in various malignancies, including skin cancer. Its role in catalyzing the N-methylation of nicotinamide and involvement in homeostasis and detoxification processes suggest its potential as a therapeutic target for melanoma treatment [118,119,120,121]. Paraoxonase-2 (*PON2*) is another notable biomarker, with significant upregulation observed in melanomas compared to control nevi. *PON2* expression correlates with important prognostic parameters, indicating its potential as a prognostic biomarker and its involvement in melanoma cell resistance to chemotherapy [122,123,124]. Moreover, the transcription factor *Nrf2* has been linked to an altered redox homeostasis in melanomas, influencing phenotype switching. While directly targeting NRF2 may require a combinatorial approach, its downstream antioxidant gene products could be explored as more effective therapeutic targets [125,126,127].

Additionally, other biomarkers such as *MITF* (microphthalmia-associated transcription factor), *IDO* (indoleamine 2,3-dioxygenase), and *MDM2* (mouse double minute 2 homolog) have shown promise in regulating melanoma cell behavior, offering potential avenues for targeted therapeutic strategies. As research progresses, the identification and validation of these biomarkers could revolutionize melanoma management, leading to improved patient outcomes and personalized treatment approaches [128,129,130,131,132,133,134,135].

## 9. Conclusions

CM and UM are distinct tumor types with significant differences in tumorigenesis, genetic alterations, metastatic dissemination pathways, and responses to therapies. Although both malignancies commonly involve abnormalities in certain chromosomal regions, the rates at which these occur differ. CM frequently exhibits abnormalities on chromosomes 1, 6, 7, 9, 10, 14, 16, and 21, while UM is characterized by chromosomal aberrations on chromosomes 1, 3, and 8. Monosomy 3, the gain of 8q, monosomy 3, and the loss of 1p36 are all significant factors associated with a poor prognosis.

In CM, approximately 80% of patients have mutations in the *BRAF*, *NRAS*, or *NF1* genes, leading to the deregulation of the *ERK* pathway. On the other hand, UM frequently harbors activating mutations in *GNAQ/11* (83%). UM also exhibits recurrent genetic mutations in *BAP1*, which plays a role in cell cycle regulation, cell identity, genome integrity, and metastasis development.

Both types of tumors have a propensity to spread, with UM mainly spreading hematogenously and showing a strong liver tropism. In contrast, CM metastasis affects the lungs, liver, bones, and brain at nearly equal rates.

Significant advancements have been made in the treatment of advanced CM, leading to improved life quality and overall survival. Primary UM diagnosis and therapies have also seen significant improvements in recent decades, and the recent approval of tebentafusp has increased life expectancy in HLA-A*02:01-positive patients with metastatic disease. Genetic, clinical, and histopathological markers have shown promise in predicting prognosis and guiding patient-tailored therapies.

The study of *NNMT*, *PON2*, *Nrf2*, *MITF*, *IDO,* and *MDM2* as potential biomarkers in melanoma holds great promise for improving diagnosis and advancing targeted therapies. Further research and exploration are needed to fully understand their roles and interactions, paving the way for novel treatments in the battle against melanoma.

Despite these advances, no efficacious treatment has been found for metastatic melanoma. Therefore, a further exploration of mutations associated with tumor growth, proliferation, metastasis, and survival is necessary to better understand their pathogeneses and metastatic mechanisms and develop more potent treatments.

## Figures and Tables

**Table 1:** Novel molecular biomarkers for targeted therapies in melanoma.

Therapeutic Target	Effectory Function	Evidence
NNMT	key enzyme involved in in nicotinamide homeostasis, biotransformation, and detoxification of xenobiotic compounds by catalyzing the N-methylation of nicotinamide	[118,119,120,121]
PON2	an intracellular enzyme functions protectively by inhibiting the generation of reactive oxygen species within the mitochondrial respiratory chain	[122,123,124]
Nrf2	transcription factor implicated in the altered redox homeostasis, which led to changes in genes related to melanoma progression, suggesting its involvement in phenotype switching	[125,126,127]
MITF	transcription factor as a key regulator of melanoma cell proliferation, survival, and invasion	[128,129]
IDO	enzyme that mediates the conversion of tryptophan to kynurenine, leading to immune suppression in the tumor microenvironment	[130,131,132]
MDM2	overexpression can lead to the inactivation of p53, promoting tumor growth and decreasing survival	[133,134,135]

## Data Availability

All information provided in this review is documented by relevant references.
